# Physiological Response of the Freshwater Mussel *Unio douglasiae* in *Microcystis aeruginosa* Bloom Waters

**DOI:** 10.1155/2022/2928235

**Published:** 2022-04-06

**Authors:** Zhun Li, Young-Hyo Kim, David C. Aldridge, Baik-Ho Kim

**Affiliations:** ^1^Biological Resource Center/Korean Collection for Type Cultures (KCTC), Korea Research Institute of Bioscience and Biotechnology, Jeongeup 56212, Republic of Korea; ^2^Department of Environmental Sciences, Hanyang University, Seoul 04763, Republic of Korea; ^3^Department of Zoology, University of Cambridge, The David Attenborough Building, Cambridge CB2 3QZ, UK; ^4^Department of Life Science and Research Institute for Natural Sciences, Hanyang University, Seoul 04763, Republic of Korea

## Abstract

In the present study, we evaluated the effects of different environments on the filtering rate (FR), mortality, and biodeposition (BD) of the freshwater mussel *Unio douglasiae* in bloom waters containing the toxic cyanobacterium *Microcystis aeruginosa*. The mean FR of 19 selected individuals (shell length, 5.0–9.8 cm) was 0.30 ± 0.03 L g^−1^ h^−1^ (range = 0.24–0.35 L g^−1^ h^−1^). Shell length was strongly correlated with both net and gross BD of mussels (*P* < 0.0001). The mean FR was higher in river water (0.405 ± 0.052 L g^−1^ h^−1^) than in lake water (0.304 ± 0.051 L g^−1^ h^−1^). In contrast, the BD of mussels was higher in RW (0.671 ± 0.609 mg g^−1^ h^−1^) than in LW (0.275 ± 0.027 mg g^−1^ h^−1^). For algal species, the FR of mussels ranged from 0.114 ± 0.024 to 0.553 ± 0.019 L g^−1^ h^−1^. The FR of *U. douglasiae* was higher in river water (mainly diatoms), whereas BD was higher in lake water (mainly *Microcystis*). *U. douglasiae* did not prefer toxic *M. aeruginosa*, which was significantly accumulated in pseudofaeces and faeces. The maximum FR of *U. douglasiae* in algal bloom water was recorded at a water temperature of 25°C and water depth of 50 cm. Moreover, the *in situ* mortality of *U. douglasiae* was strongly affected by water temperature and nitrogen concentration. Overall, *U. douglasiae* can enhance water quality in eutrophic areas by removing dominant cyanobacteria, although its removal efficiency depends on environmental parameters and site of introduction.

## 1. Introduction

Freshwater mussels are benthic molluscs serving pivotal functions in both lentic and lotic ecosystems, such as the removal of different-sized sestons, including phytoplankton [1–3]; biodeposition (BD) of particulate organic matter, such as faeces and pseudofaeces [4–6]; and release of inorganic nutrients [7–9]. Many laboratory and *in situ* studies have proven the potent filtering abilities of mussels [9–13], highlighting their potential use for improving water quality [13, 14]. Therefore, many attempts have been made to improve water quality using freshwater unionids [15, 16]. Freshwater unionids can alter cyanobacterial densities by promoting bloom formation [17–19]. Nevertheless, the effects of freshwater unionids on cyanobacterial densities remain contradictory. Various studies have documented that filtration by freshwater unionids decreases cyanobacterial densities [20, 21], whilst other studies found that the presence of freshwater unionids in invaded ecosystems increased cyanobacterial densities [17, 18, 22, 23]. However, most previous studies on bivalve filtration were performed with limited laboratory cultures, whilst few studies have explored using field bloom waters.

Cyanobacterium overpopulation is a global problem, particularly in Korean freshwater ecosystems, due to bloom formation and toxin production. The mass mortality of aquatic organisms in aquatic ecosystems is often ascribed to cyanobacteria [24]. In Korean freshwater environments, cyanobacteria and diatoms are the major bloom-forming microalgae. Amongst these, toxic and nontoxic *Microcystis aeruginosa* are the most dominant species of bloom-forming cyanobacteria. Toxic *M. aeruginosa* produces microcystins, a group of neurotoxins posing a major threat to drinking and irrigation water supplies as well as the environment. Gazulha et al. [25] showed that freshwater unionids preferred nontoxic algae as their prey (i.e., the diatom *Nitzschia palea* and nontoxic *M. aeruginosa*) and rejected toxic *M. aeruginosa* through pseudofaeces. Although some mussels can simply close their shells to reject harmful algae, they must open the shell for filtering water to breath. A long-term grazing experiment revealed no negative effects of toxic *Microcystis* on the filtration capability and survival of the golden mussel, suggesting that factors other than toxicity drive selective feeding in this species [26].

To date, 11 members of the Unionidae family have been identified in Korea [27, 28], including *Unio douglasiae sinuolatus* von Martens (1905), *Lanceolaria grayana* Lea (1834), *Solenaia triangularis* Heude (1885), *Cristaria plicata* Leach (1815), *Lamprotula coreana* von Martens (1905), *Lamprotula leai* Griffith & Pidgeon (1834), *Anodonta arcaeformis* Heude (1877), *Anodonta arcaeformis flavotincta* von Martens (1905), and *Anodonta woodiana* Lea (1834). Of these, *U. douglasiae*, a representative species from Korea, has been used as a cooking material by the older Korean population over the past 500 years, although few people consume foods containing its muscle portion due to hard texture and present-day sanitation methods. *U. douglasiae* is the most widely distributed from freshwaters to brackish waters across Asia, including Korea, China, Japan, Vietnam, and Taiwan [29, 30]. In Korea, *U. douglasiae* is commonly used to construct “*Bo*”—a shapeless structure made with a mix of pebbles, dry grass, dead leaves, and soil—which is used to trap water for drinking, irrigation, and recreational use as well as to support various fisheries and fish spawning activities. Historically, dam construction using *Bo* was rather popular; however, at present, Bo is primarily used at a small scale for various purposes, such as to construct concrete dams on lakes. Unfortunately, the construction of these dams heavily pollutes and eutrophicates water bodies through the input of wastewater containing contaminants, such as agrochemicals and heavy metals [31, 32], ultimately inhibiting the growth of various aquatic organisms.

To date, few studies have explored the physiological responses of freshwater mussels in *Microcystis* bloom waters. Therefore, the present study evaluated the effects of different dissolved oxygen (DO) concentrations and water depths on the filtering rate (FR), mortality, and BD of *U. douglasiae* in *M. aeruginosa* bloom water. Furthermore, we compared the FR and BD of *U. douglasiae* feeding on different algal species, including nontoxic and toxic *Microcystis aeruginosa* (NIES-101 and NIES-298), *Anabaena flos-aquae* (NIER-30010), *Oscillatoria sancta* (NIER-10019), *Chlorella vulgaris* (UTEX-265), *Selenastrum gracile* (KCTC-AG10009), and *Nitzschia palea* (NIES-489).

## 2. Materials and Methods

### 2.1. Mussel Collection and Maintenance

*Unio douglasiae* was collected by towing a dredge or by hand from a reach of the North Han River (35°56′02′′ to 35°54′13′′N, 126°47′07′′ to 126°45′43′′E) between Lake Ilgam and Lake Cheongpyeong from 21 to 28 October 2010. The stream from which the mussels were collected has the average width of 250 m, depth of 4 m, average current of 50 cm s^−1^, and sediment composition of pebble–clay–silt (~2 : 3 : 5). The collected mussels were thoroughly washed with a brush to remove debris and carefully transferred to the mussel acclimatisation system (MAS). The system comprised three tanks (T1, T2, and T3) in the laboratory, which were automatically maintained at the following parameters: temperature of 18–20°C, flow rate of <2 L min^−1^, light intensity of 30 *μ*mol m^−1^ s^−1^, and 14 h light : 10 h dark photo-cycle. All tanks were made of the same material, polyvinylchloride (PVC), and had the same structure of a cylindrical vessel with an ellipsoidal base (150 L in volume and 40 cm in depth). T1 was maintained as the natural water tank, which was supplied twice daily with surface water (20 L d^−1^) from the eutrophic Lake Ilgam (35°56′02^″^N, 126°47′07^″^E). This lake hosts cyanobacterial blooms, mainly formed by *M. aeruginosa*, every summer [33, 34]. In T2, naturally occurring stream sediments were added, and mussels were housed at the density of 80 individuals·m^−2^. T3 received the water that was passed through T2 and was continuously aerated with a carbon stone, filtered with a commercial sanitary gauge, and finally returned to T1 using an electric pump. Mussels in T2 were monitored daily by visual examination for viability. Mussels with opened shells, immobility, and no green mass or aggregates near the exhalant siphon were considered dead and immediately removed. All mussels were acclimatised over 2 months in the MAS. The mussel mortality rate was approximately 1.0 individual every 2 months. All mussels were starved for approximately 2–3 days before the start of the experiment, and they were immediately returned to the animal collection stream after completion of experiments.

### 2.2. Preparation of Algal Bloom Water

For experiments, algal bloom water (ABW) was prepared using surface water and sediment from the same lake as above [33, 34]. Currently, the lake is being sustained by groundwater and rainwater, with a water residence time of approximately a year. Based on 3 years of data, the annual mean chlorophyll-*a* (chl-*a*) concentration is 41.0 *μ*g L^−1^, total phosphorus concentration is 0.10 *μ*g L^−1^, and total nitrogen concentration is 1.73 mg L^−1^ [33]. Every year, water clarity of the lake is low (0.51 in Secchi depth) due to the presence of dense cyanobacterial blooms between early summer and late autumn as well as of diatoms and green algae during winter.

On 15 July 2010, a bloom occurred. Surface water (>50 cm in depth) and sediment (>5 cm of soil depth) samples were simultaneously collected from the lake using a 5 L Van Dorn sampler and an Ekman dredge and transferred to several glass aquaria (45 × 50 × 120 cm^3^) in the laboratory. All aquaria were maintained at 24°C under cool-white fluorescent light (45 *μ*E m^−2^·s^−1^) and 14 h light : 10 h dark photo-cycle. Before the experiment, ABW was filtered with a plankton net (64 *μ*m) to remove zooplankton, and the algal density, suspended solids (SS), and chl-*a* were measured. chl-*a* concentration was determined using a spectrophotometer (DU 800, Beckman Coulter, Inc., USA) after passing the subsamples through GF/C filters (pore size = 1.2 *μ*m, Whatman, UK), followed by extraction in 90% acetone for 24 h [35]. SS content was calculated as the difference in the concentration of particulates filtered with GF/C filters (pore size = 1.2 *μ*m, Whatman) between the control and test vessels at each sampling time [35]. For phytoplankton quantification, 10 mL subsamples were collected from the aquaria and fixed in 1% (*v*/*v*) Lugol's iodine solution. Algal cells were counted under an inverted microscope with a Sedgwick–Rafter chamber [36]. Species were identified through examination under a light microscope (Olympus, Tokyo, Japan) as described by Akiyama et al. [37].

### 2.3. FR and BD of Mussels

To measure the FR and BD of *U. douglasiae*, the length, height, and wet weight of the mussels were measured. First, ash-free dry matter (AFDM) of the mussels was calculated with linear regression using shell length and AFDM of the sacrificed mussels. AFDM was measured according to the method described by Kim et al. [34]. Briefly, the body (mantle, muscles, and digestive organs) of each mussel was carefully dissociated using a knife. The harvested body parts were weighed, transferred to fire-resistant vessels, and heated in a furnace at 500°C for 30 min. After desiccation of the wet mussel body at 70°C for 30 s in a dry oven, the parts were weighed again. AFDM of each mussel was calculated based on the difference in dry weight (mg) before and after heating. Of note, shell length of mussels was closely related to their AFDM (*r*^2^ = 0.850, *n* = 59, *P* < 0.0001).

The FR of *U. douglasiae* was calculated as the difference in chl-*a* concentration between the control and test vessels at each sampling time using Coughlan's formula [38] as follows:

(1) $$\begin{array}{c} \text{FR}\left(Lg^{-1}h^{-1}\right)=\frac{V}{g}\times \frac{ln\left(C1/C2\right)}{t}, \end{array}$$

where *V* is the volume of the experimental vessel, *g* is the AFDM of the mussels, *C*1 and *C*2 are the chl-*a* (*μ*g L^−1^) concentrations in the control and test vessels, respectively, and *t* is the duration of the experiment (h).

The BD of *Unio douglasiae* was determined as the difference in SS (mg L^−1^) between the control and test vessels at each sampling time as follows [9]:

(2) $$\begin{array}{c} \text{BD}\left(mgg^{-1}h^{-1}\right)=\frac{V}{g}\times ln\frac{\left(S1/S2\right)}{t}, \end{array}$$

where *V* is the volume of the experimental vessel, *g* is the AFDM of the mussels, *S*1 and *S*2 are the SS (mg L^−1^) concentrations in the control and test vessels, respectively, and *t* is the duration of the experiment (h).

### 2.4. Feeding Experiment

Feeding experiments were conducted to assess the effects of shell length, mussel density, prey species and concentration, water temperature, DO, and water depth of *U. douglasiae* (Table 1). Except in the experiment assessing the effects of prey species, ABW was used to provide naturally occurring prey. The experiments were set up in 3 L PVC aquaria at a water temperature of 23–24°C under cool-white fluorescent light (40–48 *μ*E m^−2^ s^−1^) and 14 h light : 10 h dark photo-cycle. The volume of the culture water was 2 L. In addition, except the experiment assessing the effects of mussel size, all experiments were performed in triplicate. Algal species were purchased from microbial culture collections (NIES, NIER, KCTC, and UTEX).

ABW was prepared using surface water and sediment from Lake Ilgam, where cyanobacterial (*M. aeruginosa*) blooms occur every summer. Before the experiment, ABW was filtered with a plankton net (64 *μ*m) to remove zooplankton. The effects of river water (RW) and lake water (LW) were explored (Tables 2 and 3).

*Animal size and density*: first, to determine the FR and BD of mussels, 19 individuals (at 0.5 individuals·L^−1^) with different shell lengths (ranging from 4.2 to 9.8 cm) were individually stocked in aquaria filled with 2 L of ABW at 23°C temperature, 8.3 mg L^−1^ DO, and 138.9 *μ*g L^−1^ chl-*a* concentration. Second, three aquaria were prepared at the mussel stocking rate of 0.5, 1.0, and 1.5 individuals·L^−1^ for the mussel density experiment. The FR and BD of mussels were measured at 0, 1, 4, and 7 h after stocking.

*Microcystis concentration*: to measure the FR and BD of the mussels according to prey concentration, six aquaria with different chl-*a* concentrations (49.4, 113.2, 199.7, 265.7, 327.4, and 409.8 *μ*g L^−1^) were prepared using ABW. The mussels (ranging from 7.3 to 9.2 cm in shell length) were introduced at the density of 0.5 individuals·L^−1^ in each aquarium. The FR and BD of mussels were measured at 0, 1, 4, and 7 h after stocking.

*Different natural prey*: to measure the FR of mussels feeding on common prey in their natural environment, RW and LW were used (Tables 2 and 3). Both RW and LW were collected on the same day (15 January 2010). Cell density was similar (2.4 × 10^4^ and 2.2 × 10^4^ cells mL^−1^, respectively) but chl-*a* concentration (47.1 and 25.4 *μ*g L^−1^, respectively) was different between RW and LW, indicating differences in the species composition of the phytoplankton community. RW was collected from the surface water of the lower region of Han River, where the water temperature was 1.35°C, pH was 8.4, and DO saturation was 131% (15 January 2015). The community was dominated by *Stephanodiscus hantzschii* and *Asterionella formosa* (97.6% of the total concentration). The total cell density was 2.05 × 10^4^ cells mL^−1^, chl-*a* concentration was 47.1 *μ*g L^−1^, total nitrogen was 6.24 mg L^−1^, and total phosphorus was 0.36 *μ*g L^−1^.

LW was collected from a small eutrophic lake (Lake Ilgam), as described earlier. The phytoplankton community was dominated by *Synedra ulna* and *Scenedesmus* sp. (79.4% of the total). The total cell density was 2.2 × 10^4^ cells mL^−1^, chl-*a* concentration was 25.4 *μ*g L^−1^, total nitrogen was 1.22 mg L^−1^, and total phosphorus was 0.06 *μ*g L^−1^. The FR and BD of mussels were measured at 0, 1, 4, and 7 h after stocking.

ABW is the surface water collected from Lake Ilgam, where *Microcystis* blooms occur every June through September. NRW is river surface water collected from the Seungsoo Bridge on Han River, which is characterised by a high density of *Stephanodiscus* every November through March of the following year.

*Different phytoplankton prey*: to compare the FR and BD of mussels feeding on different algae, the following seven species were used (Table 4): four cyanobacteria, namely, nontoxic *M. aeruginosa* (NIES-101), toxic *M. aeruginosa* (NIES-298), *Anabaena flos-aquae* (NIER-30010), and *Oscillatoria sancta* (NIER-10019); two green algae, namely, *Selenastrum gracile* (KCTC-AG10009) and *Chlorella vulgare* (UTEX-265); and the diatom *Nitzschia palea* (NIES-489). Under a 12 h light : 12 h dark photo-cycle and cool-white fluorescent light (100-150 *μ*mol m^−2^ s^−1^), the four cyanobacterial, two green algal, and one diatom species were cultured at 25°C and 20°C, respectively. Due to differences in algal shape, we used the chl-*a* concentration as the unit of algal prey. The FR and BD of mussels were measured at 0, 1, 4, and 7 h after stocking.

ABW: algal bloom water (see the text for details); NIES: National Institute for Environmental Studies, Japan; NIES-298: toxic *Microcystis aeruginosa*; NIES-101: nontoxic *Microcystis aeruginosa*; NIER: National Institute for Environmental Research, Korea; KCTC: Korean Collection for Type Cultures, Korea; UTEX: University of Texas, USA.

*Different environmental conditions*: first, to examine the effect of temperature on the FR and BD of mussels, four aquaria were maintained at different temperatures (5, 15, 25, and 35°C) using a temperature controller (B2-1000M, JEIOTECH, J-MP1, JISICO). Each 9 L aquarium was filled with ABW, corresponding to the chl-*a* concentration of 49.4 *μ*g L^−1^. Mussels (5.0–7.9 cm in shell length) were introduced at the density of 0.3 individuals·L^−1^ in each aquarium. The FR and BD of mussels were measured at 0, 1, 4, and 7 h after stocking.

Second, to understand the effect of DO on the FR and BD of mussels, three different DO concentrations (0.5, 4.5, and 9.0 mg L^−1^) were used. Mussels (6.5–7.1 cm in shell length) were stocked at a density of 0.5 individuals·L^−1^ in each 3 L PVC aquarium filled with 2 L of ABW, corresponding to the chl-*a* concentration of 190 *μ*g L^−1^. DO concentration of the experimental aquaria was adjusted with nitrogen gas. The FR and BD of mussels were measured at 0, 1, 4, 7, and 11 h after stocking.

Third, the FR of mussels at different depths was measured in a cylindrical polyacrylamide chamber (height = 110 cm, diameter = 27 cm) filled with ABW, corresponding to the chl-*a* concentration of 87.8 *μ*g L^−1^. To mount the mussels at each depth, circular plates with stainless steel grids were installed. Mussels were stocked at the density of 0.7 individuals·L^−1^ in each layer (20, 50, and 80 cm) at 20°C temperature under cool-white fluorescent light (70 *μ*E m^−2^·s^−1^) and 14 h light : 10 h dark photo-cycle. Subsamples were collected at 0, 4, 8, 12, 18, 30, 48, and 72 h after stocking. Note that BD was not measured to avoid disturbing the sampling.

*In situ mortality*: over 92 days, the mortality rate (individuals·d^−1^) of mussels was measured at different depths (20, 50, and 80 cm) in the shallow region of the eutrophic Lake Shingu [39]. Briefly, a small mesocosm (1 × 1 × 1.5 m^3^) was constructed to stock the mussels in the watershed reservoir. Mussels (*n* = 20, shell length = 6.0 ± 1.0 cm) were stocked into the lattice space (40 × 30 × 15 cm^3^) with stainless steel grids (1 × 1 cm^2^) to protect against predators, such as large fish. Mussels with opened shells were identified as dead and removed from the cages immediately upon observation. To understand the association of mussel survival with abiotic variables and environmental factors, water temperature, DO, electric conductivity, pH, turbidity, SS, and ammonia concentration were measured weekly at the same time.

### 2.5. Data Analysis

Multivariate statistical analyses were performed to compare mussel mortality and physicochemical parameters. Mussel mortality and environmental factors were normalised by logarithmic transformation before analysis. Detrended correspondence analysis (DCA) and redundancy analysis (RDA) were performed using CANOCO version 4.55 for Windows [40]. DCA was used to test the characteristics of variability in mortality. The length of the first DCA gradient was 1.0 standard deviation for our data set. This result justified further use of RDA. The significance of each environmental factor was determined using the Monte Carlo test based on 499 unrestricted permutations. Pearson's correlation analysis of means was used to examine the link between mussel mortality and physicochemical parameters. The correlation between environmental factors and mussel mortality was considered significant at *P* < 0.05.

## 3. Results

### 3.1. Filtration Rate and Mussel Size

**Figure 1:**
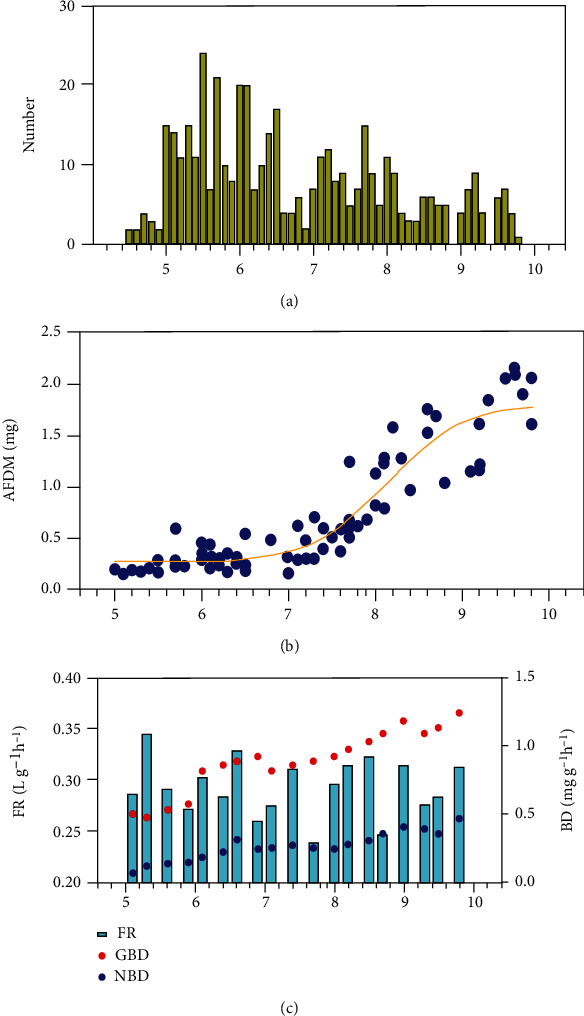
Size distribution, weight (ash-free dry-matter, AFDM), filtration rate (FR), gross biodeposition (GBD), and net biodeposition (NBD) of the freshwater mussel *Unio douglasiae* in algal bloom water (ABW). (a) Size distribution of mussels (*n* = 435 individuals). (b) AFDM of randomly selected and sacrificed mussels (*n* = 83 individuals). (c) Filtration rate and biodeposition of mussels (*n* = 19 individuals). See the text on ABW for more details.

The shell length of *U. douglasiae* (*n* = 435) ranged from 4.5 to 9.8 cm (mean = 7.15 ± 1.6 cm) and showed a skewed distribution towards younger mussels (5.5–6.5 cm in shell length; Figure 1(a)). Mussel weight (as AFDM) was closely related to shell length (*n* = 83, *Y* = 0.01693^1/2.047*X*^, *r*^2^ = 0.861, *P* < 0.0001), although there were no significant differences in body weight between mussels with a shell length of 5.0 and 7.0 cm (Figure 1(b)). The mean FR of 19 selected individuals (5.0–9.8 cm in shell length) was 0.30 ± 0.03 (range = 0.24–0.35) L g^−1^ h^−1^, although these values were not correlated with shell length (*P* > 0.5). Shell length was strongly correlated with both net (NBD; *r*^2^ = 0.883, *P* < 0.0001) and gross BD (GBD; *r*^2^ = 0.863, *P* < 0.0001) of mussels (Figure 1(c)).

### 3.2. Filtration of Different Types of Prey

**Figure 2:**
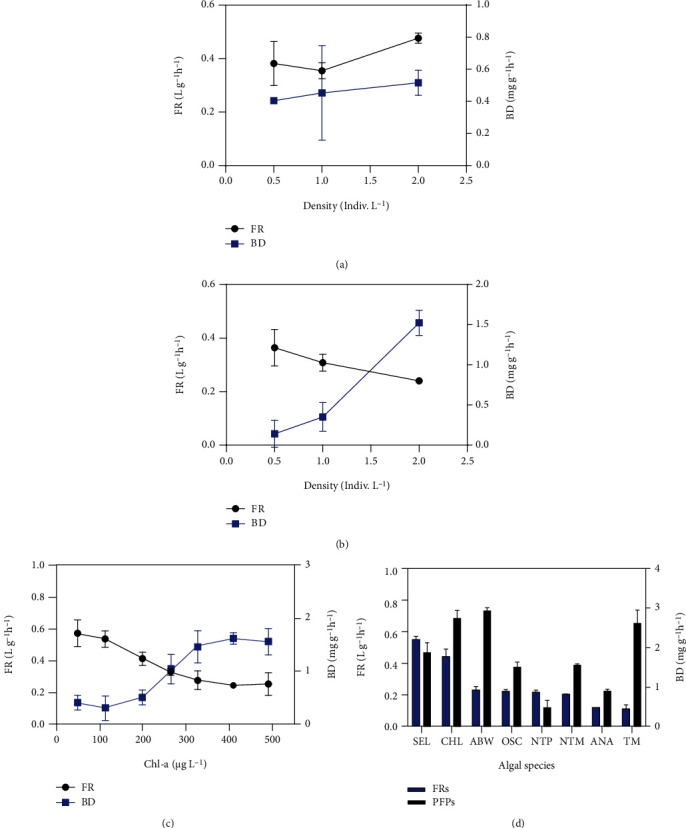
Filtration rate (FR) and biodeposition (BD) of *Unio douglasiae* at different densities (0.5, 1.0, and 2.0 individulas·L^−1^) in (a) river water (RW) and (b) lake water (LW). FR and BD of mussels at (c) six concentrations of chlorophyll-*a* and with (d) eight different algal species as prey. SEL: *Selenastrum gracile* (KCTC-AG10009); CHL: *Chlorella vulgare* (UTEX-265); ABW: algal bloom water; OSC: *Oscillatoria sancta* (NIER-10019); NTP: *Nitzschia palea* (NIES-489); NTM: nontoxic *Microcystis aeruginosa* (NIES-101); ANA: *Anabaena flos-aquae* (NIER-30010); TM: toxic *Microcystis aeruginosa* (NIES-298).

With increase in density, the FR of mussels gradually decreased in LW but slightly increased in RW. The mean FR was higher in RW (0.405 ± 0.052 L g^−1^ h^−1^; range = 0.355–0.477 L g^−1^ h^−1^) than in LW (0.304 ± 0.051 L g^−1^ h^−1^; range = 0.240–0.368) (Figure 2(a)). In contrast, the BD of mussels was higher in RW (0.671 ± 0.609 mg g^−1^ h^−1^; range = 0.140–0.525 mg g^−1^ h^−1^) than in LW (0.275 ± 0.027 mg g^−1^ h^−1^; range = 0.243–0.310 mg g^−1^ h^−1^) (Figure 2(b)).

At different concentrations of ABW, FR (0.244 ± 0.072 to 0.57 ± 0.09 L g^−1^ h^−1^) decreased but BD (0.30 ± 0.23 to 1.56 ± 0.30 mg g^−1^ h^−1^) gradually increased (Figure 2(c)) with increase in prey concentration, indicating an inverse correlation between prey concentration and mussel FR.

For algal species, the FR of mussels ranged from 0.114 ± 0.024 to 0.553 ± 0.019 L g^−1^ h^−1^ (Figure 2(d)). Overall, the mussels showed a higher FR for green algae (>0.40 L g^−1^ h^−1^) than for cyanobacteria and diatoms (<0.30 L g^−1^ h^−1^). A relatively low FR was recorded for cyanobacteria, whilst ABW (2.930 mg g^−1^ h^−1^) and NIES-298 (2.616 mg g^−1^ h^−1^) showed higher BD.

### 3.3. Filtration and In Situ Mortality in Different Environments

**Figure 3:**
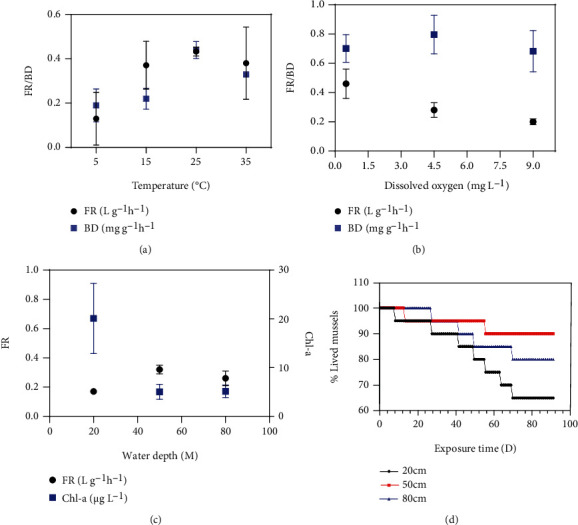
Filtration rate (FR) and biodeposition (BD) of *Unio douglasiae* under different environmental factors: (a) water temperature, (b) dissolved oxygen, and (c) water depth in aquaria containing algal bloom water (ABW). (d) *In situ* mortality of mussels in eutrophic Lake Shingu over 91 days.

At temperatures of 5 to 35°C, the FR and BD of *U. douglasiae* ranged from 0.13 ± 0.12 to 0.43 ± 0.02 L g^−1^ h^−1^ and 0.19 ± 0.07 to 0.44 ± 0.04 mg g^−1^ h^−1^, respectively (Figure 3(a)), with the maximum values recorded at 25°C. The FR of mussels was higher at a lower DO concentration (0.20 ± 0.02 to 0.46 ± 0.10 L g^−1^ h^−1^) and gradually decreased with increasing DO concentration. In contrast, there was no correlation between BD (0.68 ± 0.14 to 0.80 ± 0.13 mg g^−1^ h^−1^) and DO concentration (Figure 3(b)). Amongst different water depths, the maximum FR of 0.32 ± 0.03 L g^−1^ h^−1^ was recorded at 50 cm and the minimum FR of 0.17 ± 0.01 L g^−1^ h^−1^ at 20 cm (Figure 3(c)).

**Figure 4:**
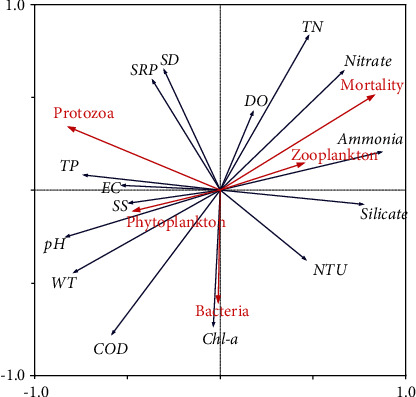
Ordination diagram generated from the redundancy analysis (RDA) showing the mortality of *Unio douglasiae* and environmental variables on RDA axes.

In the shallow eutrophic lake, the *in situ* mortality rate of *U. douglasiae* gradually increased with increasing time (up to 92 days), decreasing water temperature, and increasing nitrogen concentration (Figure 3(d)). The RDA ordination diagram is shown in Figure 4. The RDA output showed that the first and second ordination axes explained 70.8% and 23% of the total variation, with eigenvalues of 47.6% and 11.1%, respectively. The mortality rate of mussels was the highest at the water depth of 20 cm (40% of the control on day 70). Amongst the tested environmental variables, the mortality rate was strongly but negatively correlated with temperature (*r* = −0.964), COD (*r* = −0.893), and pH (*r* = −0.832), whilst strongly and positively correlated with nitrate (*r* = 0.875), total nitrogen (*r* = 0.831), and ammonia (*r* = 0.818) concentration (Table 5).

## 4. Discussion

### 4.1. Filtration of Unionid Mussels

Physiological and experimental conditions are known to affect the FR of bivalves against prey [41–44]. Even amongst mussels of similar shell sizes (60 mm), various values of FR have been reported worldwide (92 mL h^−1^ [45], 386 mL h^−1^ [46], 462 mL h^−1^ [45], and 1030 mL h^−1^ [47]). In British mussels, McIvor [45] demonstrated that a lower FR originated from the lack of optimal experimental conditions. In fact, to the best of our knowledge, only one European study has reported a high FR, albeit using a small number of replicates [45]. Presumably, a higher FR can only be induced under optimal conditions rather than by different measurement methods [42, 48]. In the present study, the FR of *U. douglasiae* ranged from 241 to 345 mL h^−1^ and strongly correlated with mussel shell length and density, prey type and concentration, algal species, water temperature, DO, and water depth. Mean FR of the 19 selected mussels (7.4 cm in shell length on average, ranging from 5.1 to 9.8 cm) was 294 mL h^−1^ in ABW (Figure 1). Furthermore, the mean FR of four mussels with a shell length of ~60 mm was 288 mL h^−1^, ranging from 272 to 303 mL h^−1^. The FR of mussels ranging in shell length between 5 and 10 cm was not correlated to shell size and growth stage. Therefore, the global variability in the FR of unionid bivalves may be attributed to differences in study design and experimental conditions, rather than individual variations within the species itself. Further studies are warranted to understand the filtration activity of various unionids worldwide against the same prey and under uniform experimental conditions.

### 4.2. Filtration of Natural Prey

The introduction of *U. douglasiae* at three densities resulted in different FRs and BDs in RW and LW (Table 3). With increasing mussel density, FR and BD were slightly increased in RW, whilst FR gradually decreased but BD clearly increased in LW (Figures 2(a) and 3(b)). Many studies have reported that prey concentration and composition are important factors determining the FR of mussels without optimal or natural conditions [42, 49–53]. However, little is known regarding the BD of *U. douglasiae* against different natural prey. In the present study, the feeding of *U. douglasiae* with the same prey species showed markedly different BD patterns; however, this was not the case for FR. A relatively low FR was recorded for cyanobacteria, whilst the higher BD was observed. We proposed that the feeding activities of *U. douglasiae* have an advantage for the expansion of habitat by carrying an alternative benthic-pelagic coupling, which supports diversity of suitable habitats of this species in Korea [20]. In the present study, the higher BD in RW may be attributed to the higher density of diatoms and green algae than in LW, where the cyanobacterium *M. aeruginosa* was dominant. Unionid mussels can produce pseudofaeces and faeces from cyanobacteria, which have a lower carbon biomass than diatoms and green algae [34]. Although whether the unionid mussel *U. douglasiae* exhibits selectivity for suspended particles in both running and standing water columns remains unclear. FR remained relatively stable, whilst BD was determined by the type and biomass of prey rather than the filtration activity of the mussels.

### 4.3. Filtration and Prey Concentration

With an increase in chl-*a* concentration, the FR of *U. douglasiae* gradually decreased but its BD gradually increased. Incidentally, both FR and BD did not decrease or increase further, after chl-*a* concentration reached 400 *μ*g L^−1^. The highest FR (580 ± 60 mL h^−1^) was induced by the lowest prey concentration (49.4 *μ*g L^−1^ chl-*a*), although whether low concentrations decrease FR remains unknown. In fact, the maximum chl-*a* concentration (490 *μ*g L^−1^) used in the present study is unusual and not often found in nature. This value was even higher than 300 *μ*g L^−1^ chl-*a* level used by McIvor [45] to measure the FR of unionid mussels. Furthermore, the species composition of prey in ABW differed from that tested previously. Unionid mussels have a threshold or optimum concentration of prey or sestons [51–53]. In addition, Hawkins et al. [51] demonstrated that FR was the highest with at moderate prey concentration, whilst extremely high or low prey concentrations decreased FR. Thus, future studies are warranted to compare FR between artificial and natural waters with cyanobacterial blooms showing low and high chl-*a* concentrations.

### 4.4. Mussels and Environmental Factors

The maximum FR of 432.8 ± 19.9 mL h^−1^ was recorded at 25°C, although there were no significant differences amongst the other tested temperatures of 15, 25, and 35°C (*P* > 0.5). At low temperatures (5°C), the low FR of mussels was accompanied by incomplete shell opening, impediment of algal ingestion, and low BD, consistent with the observations reported by Jorgensen et al. [54, 55]. Bivalve mussels require a specific optimum temperature that induces the maximum FR [53, 56, 57]. Korean streams are characterised by seasonality in temperature, with values of 0–10°C during winter, 10–20°C during spring and autumn, and 21–31°C during summer [58]. Except for those in some regions in southern Korea, most streams are ice-covered throughout the winter. In the MAS, many mussels often died when the temperature dropped below 7–8°C, suggesting that *U. douglasiae* cannot survive well in winter. Except the lowest temperature, neither thermal stress nor relatively high temperatures of 30–32°C [59, 60] strongly affect the FR of bivalve mussels in many Korean streams.

In the present study, the lowest DO concentration (0.5 mg L^−1^) induced the maximum FR of 460.0 ± 100.0 mL h^−1^, which was approximately two times higher than that observed at other DO concentrations (4.5 and 9.0 mg L^−1^). FR at low DO concentrations (or hypoxia) increases due to enhanced respiration rather than foraging [61–63]. The high FR at low DO concentrations, often accompanied by high temperatures, ultimately enhances the mortality of mussels by increasing algal toxin production and ammonia concentration [64–67]. In our 3-day experiment, no mussel died in ABW at 25°C. In contrast, bivalve mussels have been reported to survive over 7 days under hypoxia by physiologically regulating or diminishing oxygen consumption at low DO concentrations, regardless of temperature [63, 68, 69].

In both *in situ* and laboratory experiments, a low water depth decreased the FR and enhanced the mortality of *U. douglasiae*. Pynnönen and Huebner [70] reported that a decrease in pH (i.e., from 7 to 5) led to the closing of the valve, reduced filtration, and ultimately enhanced the mortality of bivalve mussels. However, in the present study, a lower depth (20 cm) was hydrologically characterised by higher temperatures and chl-*a* concentrations than greater depths (50 and 80 cm). Some bivalves can open or close their valves depending on the presence of contaminants in the water [71, 72] and the concentration of contaminants deposited within their tissues [52, 53]. Based on our results, the gradual increase in the mortality of mussels was strongly correlated with nitrogen compounds at low depths over 92 days. High ammonia concentrations, which are often accompanied by high temperatures and hypoxia [65, 67], markedly increase the mortality of mussels even in flowing systems [34]. However, compared with that in the previous laboratory experiments, ammonia concentration in the lake was approximately 10 to 20 times lower, regardless of mussel stocking. In addition, no hypoxia was noted throughout the study period. Furthermore, the possible effects of algal toxins warrant attention. *M. aeruginosa* blooms rarely occur in this small reservoir [38, 73]; however, the full spectrum of toxicity of contaminants to animals remains unknown. Nonetheless, during the present study, we rarely noted cyanobacterial blooms. Thus, to identify the cause of mortality of mussels at low depths, additional variables (except those mentioned previously), such as light shock, culture time, and harmful organisms, such as bacteria and viruses, must be considered. Whilst using mussels as a tool to restore water quality through the removal of SS, including dense phytoplankton, mussel stocking close to the surface of the lake should be avoided. In addition, future studies should examine the role of environmental factors in mussel mortality and verify the importance of water quality (i.e., heavy metals or microcystins) rather than the type of prey, which can be affected by laboratory conditions.

## 5. Conclusions

Based on the effects of different environmental factors on the FR, mortality, and BD of *U. douglasiae* in toxic *M. aeruginosa* bloom waters, the key findings of the present study can be summarised as follows:

1. Although larger mussels accumulated more phytoplankton (mainly *Microcystis*), the FR of individual mussels did not change with increase in their size
2. The FR of *U. douglasiae* was higher in RW (mainly diatoms), whilst its BD was higher in LW (mainly *Microcystis*)
3. *U. douglasiae* did not prefer toxic *M. aeruginosa*, which was highly accumulated in pseudofaeces and faeces

The *in situ* mortality of *U. douglasiae* was strongly affected by water temperature and nitrogen concentration.

## Figures and Tables

**Table 1:** Experimental design for the freshwater mussel *Unio douglasiae*. Filtration rate (FR) and biodeposition (BD) as faeces and pseudofaeces of *U. douglasiae* under different conditions over 7 hours of cultivation.

Variables	Mussel size	Mussel density	Type of prey	Concentration of prey	Temperature	Dissolved oxygen	Water depth
Shellfish tested/replicates	19/1	3/3	2/3	7/3	4/3	3/3	3/3
Volume (L)	2.0	2.0	2.0	2.0	9.0	2.0	57.0
Mussel size (cm)	4.2–9.8	6.3–9.1	6.3–9.1/5.5–8.5	7.3–9.2	5.0–7.9	6.5–7.1	6.5–8.9
Mussel density (individuals·L^−1^)	0.5	0.3, 1.0, 2.0	1.0	0.7	0.3	1.5	0.1
Type of prey	ABW	ABW	RW/LW	ABW	ABW	ABW	ABW
Concentration of prey (chl-*a*, *μ*g·L^−1^)	138.9	138.9	47.1/25.4	49.4, 113.2, 199.7, 265.7, 327.4, 409.81	49.4	190.0	87.8
Water temperature (°C)	23	23	1.4/4.3	23	5, 15, 25, 35	23	21.5
Dissolved oxygen (mg L^−1^)	7–8	7–8	10–12/8–9	7–8	7–8	0.5, 4.5, 9.0	8.5–8.1
Water depth (cm)	7–9	7–9	7–9	7–9	10–12	7–9	20, 50, 80
pH	8.3	8.3	8.4/8.1	8.3–9.8	8.5	8.3–8.6	8.2

**Table 2:** Environmental variables at the surface of river water (RW) and lake water (LW) (15 January 2010).

Variables	RW	LW
Sampling location	37°31′30^″^N, 127°04′30^″^E	37°31′50^″^N, 127°05′10^″^E
Sampling time	10:00 AM	11:00 AM
Temperature (°C)	1.35	4.43
Conductivity (*μ*S·cm^−1^)	108	316
Salinity (‰)	0.10	0.25
Dissolved oxygen saturation (%)	131	99
pH	8.4	8.1
Turbidity	1.7	7.8
Total nitrogen (mg·L^−1^)	6.24	1.22
Total phosphorous (mg·L^−1^)	0.36	0.06
Cell density (10^4^ cells·mL^−1^)	2.37	2.20
Carbon content (mg C·cell^−1^)	15.56	1.77
chl-*a* (*μ*g·L^−1^)	47.1	25.4

**Table 3:** Algal removal activity (ARE) of *Unio douglasiae* in river water (RW) and lake water (LW). ARE (%) = (1 − treatment/control) × 100. Zero and negative values indicate the absence of and increase in algal density, respectively, following mussel introduction. Asterisks indicate new detectable phytoplankton observed following mussel introduction.

Water type	Species observed	ARE (%)		
		0.3 individuals·L^−1^	1.0 individuals·L^−1^	2.0 individuals·L^−1^
RW	*Anabaena flos-aquae*	∗	∗	∗
	*Ankistrodesmus falcatus*	0.0	-133.3	0.0
	*Asterionella formosa*	65.2	11.4	15.6
	*Cryptomonas ovata*	∗	∗	∗
	*Diatoma vulgare*	40.0	40.0	100.0
	*Microcystis aeruginosa*	100.0	96.6	100.0
	*Navicula* sp.	100.0	100.0	100.0
	*Nitzschia holisatica*	∗	∗	∗
	*Pinnularia major*	0.0	0.0	100.0
	*Stephanodiscus hantzschii*	78.5	51.4	68.4
	*Synedra ulna*	∗	∗	∗

LW	*Aulacoseira granulata*	100.0	36.4	100.0
	*Chroococcus turgidus*	58.1	-3.2	-35.5
	*Cryptomonas ovata*	92.9	87.5	46.4
	*Diatoma vulgare*	61.1	30.6	55.6
	*Melosira varians*	100.0	83.3	100.0
	*Microcystis aeruginosa*	∗	∗	∗
	*Navicula* sp.	100.0	100.0	0.0
	*Pediastrum simplex*	100.0	-87.5	-100.0
	*Scenedesmus acuminatus*	100.0	-140.0	-180.0
	*Scenedesmus quadricauda*	53.8	68.4	57.1
	*Synedra ulna*	24.2	17.7	29.1
	*Tetraedron caudatum*	0.0	11.1	33.3

**Table 4:** Algal species as prey and chemical media used in the present study. Mean shell size and ash-free dry matter (AFDM) of three mussels used in each experiment are shown. chl-*a* indicates the initial concentration of chlorophyll-*a* in the experimental vessels. All experiments were completed in triplicate, and the mussels were stocked at 1 individuals·L^−1^.

Algal strains	Medium	Size (cm)	AFDM (mg)	chl-*a* (mg·L^−1^)
ABW	—	7.17 ± 0.29	0.67 ± 0.29	95.53
*Microcystis aeruginosa* NIES-298	BG-11	6.73 ± 0.40	0.53 ± 0.13	98.05
*Microcystis aeruginosa* NIES-101	BG-11	7.00 ± 0.00	0.61 ± 0.00	103.35
*Anabaena flos-aquae* NIER-30010	BG-11	7.17 ± 0.42	0.67 ± 0.14	95.80
*Oscillatoria sancta* NIER-10019	BG-11	7.00 ± 0.00	0.61 ± 0.00	97.07
*Selenastrum gracile* KCTC-AG10009	Allen	7.30 ± 0.26	0.72 ± 0.09	102.33
*Chlorella vulgaris* UTEX-265	Allen	7.10 ± 0.17	0.65 ± 0.06	102.81
*Nitzschia palea* NIES-489	DM	7.00 ± 0.50	0.62 ± 0.17	103.51

**Table 5:** Correlation coefficients (*r*) between the mortality of *Unio douglasiae* and environmental variables at the depth of 20 cm, at which the highest mortality was observed. Variables are listed in the order of decreasing absolute values. Statistical significance is denoted by asterisks (^∗^*P* < 0.05 and ^∗∗^*P* < 0.01, sequential Bonferroni test).

Variables	*R*
Water temperature	-0.964^∗∗^
Chemical oxygen demand	-0.893^∗∗^
pH	-0.832^∗^
Protozoa density	-0.571
Total phosphorus	-0.529
Phytoplankton density	-0.464
Chlorophyll-*a*	-0.357
Bacterial density	-0.331
Suspended solids	-0.329
Electric conductivity	-0.284
Nitrate	0.875^∗^
Total nitrogen	0.831^∗^
Ammonia	0.818^∗^
Silicate	0.567
Dissolved oxygen	0.444
Zooplankton density	0.249
Turbidity	0.108
Soluble reactive phosphorus	0.093
Transparency	0.028

## Data Availability

Data is available on request from the authors.
